# Muscle MRI Findings in Childhood/Adult Onset Pompe Disease Correlate with Muscle Function

**DOI:** 10.1371/journal.pone.0163493

**Published:** 2016-10-06

**Authors:** Sebastián Figueroa-Bonaparte, Sonia Segovia, Jaume Llauger, Izaskun Belmonte, Irene Pedrosa, Aída Alejaldre, Mercè Mayos, Guillermo Suárez-Cuartín, Eduard Gallardo, Isabel Illa, Jordi Díaz-Manera, Spanish Pompe Study Group

**Affiliations:** 1 Neuromuscular Disorders Unit. Neurology Department. Hospital de la Santa Creu i Sant Pau. Universitat Autònoma de Barcelona, Spain; 2 Centro de Investigación en Red en Enfermedades Raras (CIBERER); 3 Radiology department. Hospital de la Santa Creu i Sant Pau. Universitat Autònoma de Barcelona, Spain; 4 Rehabilitation and physiotherapy department. Hospital de la Santa Creu i Sant Pau. Universitat Autònoma de Barcelona, Spain; 5 Respiratory diseases department. Hospital de la Santa Creu i Sant Pau. Barcelona. Universitat Autònoma de Barcelona, Spain; Katholieke Universiteit Leuven, BELGIUM

## Abstract

**Objectives:**

Enzyme replacement therapy has shown to be effective for childhood/adult onset Pompe disease (AOPD). The discovery of biomarkers useful for monitoring disease progression is one of the priority research topics in Pompe disease. Muscle MRI could be one possible test but the correlation between muscle MRI and muscle strength and function has been only partially addressed so far.

**Methods:**

We studied 34 AOPD patients using functional scales (Manual Research Council scale, hand held myometry, 6 minutes walking test, timed to up and go test, time to climb up and down 4 steps, time to walk 10 meters and Motor Function Measure 20 Scale), respiratory tests (Forced Vital Capacity seated and lying, Maximun Inspiratory Pressure and Maximum Expiratory Pressure), daily live activities scales (Activlim) and quality of life scales (Short Form-36 and Individualized Neuromuscular Quality of Life questionnaire). We performed a whole body muscle MRI using T1w and 3-point Dixon imaging centered on thighs and lower trunk region.

**Results:**

T1w whole body muscle MRI showed a homogeneous pattern of muscle involvement that could also be found in pre-symptomatic individuals. We found a strong correlation between muscle strength, muscle functional scales and the degree of muscle fatty replacement in muscle MRI analyzed using T1w and 3-point Dixon imaging studies. Moreover, muscle MRI detected mild degree of fatty replacement in paraspinal muscles in pre-symptomatic patients.

**Conclusion:**

Based on our findings, we consider that muscle MRI correlates with muscle function in patients with AOPD and could be useful for diagnosis and follow-up in pre-symptomatic and symptomatic patients under treatment.

**Take home message:**

Muscle MRI correlates with muscle function in patients with AOPD and could be useful to follow-up patients in daily clinic.

## Introduction

Childhood/adult onset Pompe disease (AOPD) is a genetic disorder caused by mutations in the *GAA* gene leading to a deficiency of the enzyme acid α-glucosidase[1]. This enzyme degrades glycogen inside the lysosomes. Glycogen accumulates in skeletal muscles of AOPD patients leading to a number of pathologic changes producing muscle weakness[2]. AOPD patients may have different clinical presentations, such as asymptomatic hyperCKemia, slowly progressive limb girdle weakness and respiratory insufficiency secondary to respiratory muscle weakness[3].

Enzyme replacement therapy with alfa-glucosidase (ERT) has changed the natural history of AOPD[4]. Published studies show that ERT improves muscle function during the first months of treatment and stabilizes clinical situation over time[5–7]. One of the main drawbacks of this treatment is the high cost of the drug per patient and year (more than 250.000$/year). Considering that the drug stabilizes function but lifelong treatment is needed, health authorities have expressed concern regarding how to measure the effectiveness of treatment and when therapy should be started[8]. Clinical guides suggest that muscle MRI could be useful to follow progression of the disease in treated patients or to monitor signs of muscle deterioration in patients with asymptomatic hyperCKemia, anticipating the onset of their treatment[9–11].

Muscle MRI is a useful tool to study the structure of skeletal muscles. There are several sequences that analyzes different aspects of muscle [12]. T1-weighted (T1w) and 3-point Dixon imaging provide information on fat infiltration in muscle[13, 14]. T1w imaging has been used to identify patterns of muscle fatty infiltration helpful for the diagnosis of muscles diseases[15, 16]. Recent studies have pointed out the utility of 3-point Dixon sequences to quantify the percentage of fatty infiltration in muscles and follow-up patients over a period of time[17, 18]. However it is not yet known whether the degree of muscle fatty infiltration correlates with muscle strength and function in AOPD patients. This question needs to be addressed prior to consider muscle MRI as a surrogate biomarker of disease progression, especially at present, when ERT has changed the natural history of the disease. To tackle this question, we performed a transversal observational study in a large group of AOPD patients comparing muscle function tests with results of T1w and 3-Point Dixon muscle imaging.

## Material and Methods

### Clinical assessment

We are currently conducting a prospective observational study following-up a cohort of 34 genetically confirmed AOPD patients at the Hospital of Sant Pau. This study is registered in the webpage ClinicalTrials.gov with the identifier NCT01914536. All patients provided written informed consent to participate in the study. The study was reviewed and approved by the Ethics Committee at Hospital de la Santa Creu i Sant Pau.

We collected data concerning epidemiological characteristics, general medical background and current clinical status at basal visit. Two physiotherapists evaluated muscle function using the following tests: 6-minutes walking test (6MWT), time to walk 10 meters, time to up-and-go, time to climb up and down 4 steps, and motor function measure-20 items scale (MFM-20). Daily living activities were studied using Activlim test and quality of life was investigated using INQoL and SF-36 questionnaires.

Muscle strength was studied using both Muscle Research Council scale (MRC) and hand-held myometry. Using MRC, which grades muscle strength from 0 (no movement) to 5 (normal strength), we evaluated neck flexion and extension, arm abduction, elbow flexion and extension, finger flexion and extension, hip flexion, extension, adduction and abduction, knee flexion and extension, and foot flexion, extension, inversion, and eversion. Trunk flexion and extension functions, which are not commonly studied using MRC, were analyzed as follows: normal movement: 5 points, ineffective muscle contraction: 3 points, no muscle contraction: 0 points. We defined a MRC total score as the aggregate of MRC scores of every single function analyzed.

Using hand-held myometry we evaluated the following muscle functions: neck flexion, arm abduction, elbow flexion and extension, hip flexion, extension, adduction, and abduction, knee flexion and extension. These studies were performed only on patient’s dominant side. A myometry score was defined as the aggregate of scores obtained with the myometer.

We obtained forced vital capacity seated (sFVC), forced vital capacity in a lying position (lFVC), maximum inspiratory pressure (MIP) and maximum expiratory pressure (MEP) using a conventional spirometry. We performed a blood analysis to all patients to test CK levels before physiotherapy assessment. The normal values of CK in our laboratory are less than 174 U/L.

### Muscle MRI

Whole body muscle MRI was performed in a Philips Achieva XR 1.5 Teslas device at Hospital de la Santa Creu i Sant Pau. Axial T1 weighted turbo spin echo images were obtained using the following acquisition parameters TR = 757 ms, TE = 17 ms, thickness = 8 mm, number of slices = 164, FOV = 530 x 530 mm, acquired voxel size = 1.6 x 2.88 mm. 3-point Dixon images were acquired in 3D with the following acquisition parameters: TR/TE = 5.78/1.8, 4 ms, flip angle = 15 degrees, FOV = 520 x 340 x 300 mm, voxel size = 1 x 1x 3 mm of both thighs and FOV = 520 x 320 x 200 mm and voxel size = 1.3 x 1.7 x 5 mm for the lower trunk. Water and fat images were automatically obtained from the Dixon acquisition. The time to obtain all the images was 45 minutes per patient.

Two observers (J.D. and J.L.) quantified fatty muscle infiltration in T1w imaging using the modified version of the Mercuri score published by the group of Dr. Fischer [19]:

- *Normal muscle appearance*: 0 points
- *Mild*: traces of increased signal intensity on the T1-weighted MR sequences: 1 point
- *Moderate*: increased T1-weighted signal intensity with beginning confluence in less than 50% of the muscle: 2 points
- *Severe*: increased T1-weighted signal intensity with beginning confluence in more than 50% of the muscle: 3 points
- *End-stage appearance*: entire muscle replaced by increased density of connective tissue and fat: 4 points

Each muscle was evaluated at both sides. A complete list of the muscles analyzed can be found in S1 Materials. We generated a muscle MRI score that was calculated adding all the values obtained from the muscles analyzed. Its value ranged from 0 (lowest value, all muscles are scored as 0) to 290 (highest value, all muscles are scored as 4).

To analyze 3-point Dixon images we chose five cross-sectional Dixon slices of thighs and lower trunk, in which muscle volume was highest. A complete list of the muscles analyzed can be found in S1 Materials. We mapped full cross-section of muscle groups or individual muscles and the fat fractional data were generated.

A single observer (S.F-B) estimated fat content in the muscles using the PRIDE platform (Philips Research Image Development Environment) which enables analysis of Dixon images. In order to calculate the fat fraction, a ROI (Region of Interest) was drawn in both the fat and water images. Fat fraction coefficient was defined as fat⁄(fat+water) where fat and water are the image intensity values over the ROI for the water and fat images respectively.

### Statistics

We used Shapiro-Wilk test to confirm that none of our measured variables were normally distributed and we used non-parametric statistic tests for the analysis.

Mann-Whitney U test was used to compare quantitative variables and Chi-square test to compare qualitative variables. To investigate whether there was a correlation between the muscle function scales and MRI findings we used Spearman’s rank correlation (coefficient reported as r). We considered the correlation was good if P was lower than 0.05 and r was 0.65 or higher. Hierarchical analysis and graphical representation as a heatmap was performed using R software version 3.1.3 as previously described[20]. Statistical studies were performed using SPSS^®^ Statistics software version 21 from IBM^®^. All data acquired from the visits and analysis of the MRIs can be found in the Supplemental Study Data Section.

## Results

### Clinical description of the cohort

We included 34 patients with a diagnosis of AOPD (Table 1). Seven patients were considered asymptomatic as they had only hyperCKemia, without any clinical symptom of muscle weakness, while 27 patients were considered symptomatic as they had muscle weakness. HyperCKemia patients were younger than symptomatic ones (21.4 y.o. vs 50.18 y.o, Mann-Whitney U test, p<0.001) (Table 2).

**Table 1:** List of patients participating in the study.

N	Gender	Age at Study (y)	Phenotype	GAA gene		CK (U/l)	ERT	Age at ERT (y)	Wheelchair dependent	Ventilation
				Mut 1	Mut 2					
1	F	50	Proximal weakness LL + axial	IVS1-13T>G	c.1076-1G>C	251	Yes	47	N	N
2	F	48	Proximal weakness UL and LL + axial + respiratory insufficiency	IVS1-13T>G IVS1-13T>G	c.2173C>T	779	Yes	39	Y	Y
3	F	26	HyperCKemia	IVS1-13T>G	c.1889-1G>A	779	No	-	N	N
4	F	63	Proximal weakness LL + axial	IVS1-13T>G	c.2600_2604delinsA	311	Yes	59	N	N
5	M	11	HyperCKemia	IVS1-13T>G	c.573C>A	276	No	-	N	N
6	F	45	Proximal weakness LL	IVS1-13T>G	c.1532C>A	322	Yes	42	N	N
7	F	51	Proximal weakness LL	IVS1-13T>G	c.236_246del	240	Yes	47	N	N
8	M	66	Axial + respiratory insufficiency	IVS1-13T>G	c.1933G>T	406	No	-	N	N
9	F	59	Proximal weakness LL	IVS1-13T>G	c.1637A>G	341	Yes	52	N	N
10	F	55	Proximal weakness LL	IVS1-13T>G	c.2173C>T	359	Yes	48	N	N
11	M	42	Proximal weakness LL + axial + respiratory insufficiency	IVS1-13T>G	c.573C>A	606	Yes	39	N	Y
12	F	31	Proximal weakness UL and LL + respiratory insufficiency	IVS1-13T>G	c.1637A>G	391	Yes	24	Y	Y
13	F	46	Proximal weakness LL	IVS1-13T>G	C.1192dupC	396	Yes	39	N	N
14	M	47	Proximal weakness LL + respiratory insufficiency	c.2173C>T	c.2173C>T	508	Yes	45	N	Y
15	M	51	Proximal weakness LL + respiratory insufficiency	IVS1-13T>G	c.1657C>T	709	Yes	45	N	Y
16	F	51	Proximal weakness UL and LL + respiratory insufficiency	IVS1-13T>G	c.1657C>T	458	Yes	46	N	Y
17	M	22	HyperCKemia	IVS1-13T>G	c.1781G>A	1268	No	-	N	N
18	M	49	HyperCKemia	c.271G>A	c.2510G>A	641	No	-	N	N
19	M	14	HyperCKemia	IVS1-13T>G	c.573C>A	660	No	-	N	N
20	F	65	Proximal weakness LL + respiratory insufficiency	c.1781G>A	c.1194+5G>A	68	Yes	64	N	N
21	F	35	Proximal weakness LL	IVS1-13T>G	c.1A>T	355	Yes	29	N	N
22	F	40	Proximal weakness LL	IVS1-13T>G	c.1889-1G>A	831	No	-	N	Y
23	F	52	Proximal weakness LL + respiratory insufficiency	c.1781G>A	c.1194+5G>A	907	Yes	45	N	N
24	M	64	Proximal weakness UL + LL + axial + respiratory insufficiency	IVS1-13T>G	c.2481+102_2646+31del	430	Yes	57	N	Y
25	M	8	HyperCKemia	IVS1-13T>G	c.1889-1G>A	1077	No	-	N	N
26	F	57	Proximal weakness LL + respiratory insufficiency	IVS1-13T>G	c.1447G>T	394	Yes	55	N	Y
27	M	46	Proximal weakness LL	IVS1-13T>G	c.1532 C>A	882	Yes	43	N	Y
28	M	51	Proximal weakness LL	IVS1-13T>G	c.1933G>T	952	Yes	51	N	Y
29	M	51	Proximal weakness LL	IVS1-13T>G	c.1933G>T	432	No	-	N	Y
30	M	43	Proximal weakness LL	IVS1-13T>G	c.1408_1410delinsTTT	317	Yes	43	N	N
31	F	54	Axial	*Not found	*Not found	275	Yes	48	N	N
32	M	42	Proximal weakness UL + LL	IVS1-13T>G	c.655G>A	886	No	-	N	N
33	F	20	HyperCKemia	IVS1-13T>G	c.1551+1G>A	928	No	-	N	N
34	M	50	Proximal weakness LL	IVS1-13T>G	c.1637A>G	492	No	-	N	Y

Patient 31: Diagnosis was confirmed by enzymatic quantification in muscle biopsy and peripheral blood lymphocytes. No mutation was found in the GAA gene. F, female. M, male

**Table 2:** Demographic, clinical and radiologic features of hyperCKemia and symptomatic Pompe patients.

	HyperCKemia (N = 7)	Symptomatic (n = 27)	Statistical significance
Demographic characteristics			
Age at study (y)	21.4 (+/-15.5)	50.1 (+/-8.9)	<0.001
Gender female	2/7 (28.6%)	16/27 (59.3%)	0.124*
Delay in diagnosis (y)	6.1(+/- 5.1)	9 (+/- 7)	0.367
Time of progression (y)	NA	15.5	NA
Strenght and functional scales			
Total MRC score	119.5 (0.7)	96 (15.5)	<0.001
Myometry score (Nm)	288.28 (137.3)	196.63 (136.1)	0.143
Time to walk 10 meters (sec)	3.22 (0.5)	7.40 (3.7)	0.006
6 minutes walking test (meters)	594 (75.6)	403.5 (147.1)	0.003
Time climb up 4 steps (sec)	1.5 (0.2)	4.7 (3.4)	0.002
Time go down 4 steps (sec)	1.4 (0.2)	3.7 (2.5)	0.022
Time to up&go (sec)	3.8 (1.5)	9.4 (8.5)	0.140
Daily live activities and quality of live scales			
Activlim	36 (6.3)	29.3 (5.9)	0.003
SF-36 (%)			0.002
INQuol	14.4	110.6	<0.001
Laboratory, respiratory and cardiac assesments			
CVF seated (%)	95.7(12.2)	75.7(22.6)	0.049
CVF lying (%)	82.3 (12.5)	64.4(27)	0.240
CPKs (U/l)	911.5 (260.9)	523.8(236.6)	0.008
Muscle MRI-T1w imaging			
Total muscle MRI Score	6 (6.8)	125.9 (46.7)	<0.001
Muscle MRI head/arms	2.1	25.3	<0.001
Muscle MRI trunk	4.9	13.7	<0.001
Muscle MRI legs	0.5	74.3	<0.001
Muscle MRI-3 point Dixon imaging			
Thighs fat fraction (%)	11.73	38.17	<0.0001
Paraspinal muscles fat fraction (%)	18.79	65.07	<0.0001

The most common clinical complaint was muscle weakness involving lower limbs. Only 20% of patients had problems raising their arms. Twelve patients used aids for walking such as cane or stick, while two patients were wheelchair bound. Exertion dyspnea was present in 60% of patients and orthopnea was present in 28%. Thirteen of 27 symptomatic patients (48.1%) were on respiratory support; which was non-invasive and nocturnal in 12 patients and invasive in 1 patient.

Twenty-two of the 27 symptomatic patients were treated with ERT, while none of the hyperCKemia patients were under ERT. The mean age at which ERT was started was 45.7 +/- 9 years old. The mean time on ERT when the tests were performed was 4.4 +/- 1.03 years.

### Analysis of muscle function

**Fig 1:**
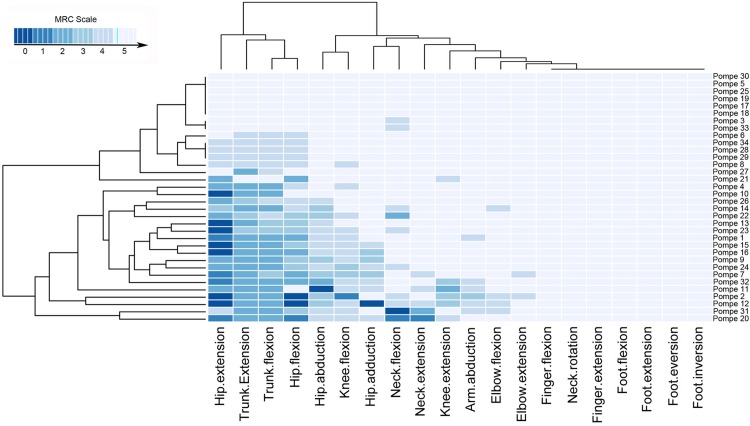
Distribution of muscle weakness in the cohort of AOPD participating in the study. The heatmap showed the MRC value for all muscles studied. Hip extension and flexion and trunk extension and flexion were the most weak impaired movements of the patients. In contrast, we did not observe distal weakness involving the lower or the upper limbs.

The MRC scale detected muscle weakness in all symptomatic patients. Hierarchical clustering analysis of the MRC scale scores showed that paraspinal, abdominal and proximal muscles of lower limbs were the weakest muscles (Fig 1). In general, hip extension was weaker than hip flexion and thigh adduction was weaker than thigh abduction. Mean muscle MRC score was 161.5 points (range 124–180). MRC score had a poor correlation with age at the time of the study (Spearman test, p:0.015; r = -0.414), with time from onset of the symptoms (Spearman test, p:0.018; r = -0.404) and with gender (Spearman test, p = 0.009, r: 0.441) and did not correlate with delay in the start of ERT (Table 3). In contrast, MRC score had a good correlation with the results of most of muscle function tests used in clinical trials such as 6MWT or the time to climb up and down four stairs, and also with scales measuring daily live activities (Activlim) or muscle function (MFM-20). Correlation between MRC score and Quality of life scales was statistically significant, but correlation coefficient was poor. We also measured strength using hand-held myometry. The mean myometry score was 196.3 points (range 55–570). There was a good correlation between the total MRC score and the Myometry score (Spearman test, p = 0.001; r: 0.667). Myometry score correlated well with muscle function tests, but this correlation coefficient was lower than correlation coefficient obtained with MRC score (Table 3).

**Table 3:** Correlation between MRC Score, Myometry Score, demographic data and muscle function tests.

	MRC Score		Myometry Score	
	Spearmantest	Correlation coefficient	Spearman test	Correlation coefficient
Demographic data				
Gender	0.009	0.441	0.01	0.553
Age at MRI	0.015	-0.414	0.049	-0.351
Time from onset of symptoms	0.018	-0.404	0.012	-0.437
Delay from onset to ERT	0.158		0.384	
Functional tests				
Time to walk 10 meters	0.0001	0.803	0.0001	0.722
6MWT	0.0001	0.708	0.0001	0.642
Time to up&go	0.083	-0.322	0.487	-0.132
Time to climb up 4 stairs	0.0001	-0.810	0.0001	-0.630
Time to go down 4 stairs	0.0001	-0.770	0.0001	-0.664
MFM-20	0.0001	0.803	0.0001	0.722
Daily live activities and Quality of life scales				
Activlim	0.0001	0.860	0.0001	0.726
SF-36	0.017	0.448	0.002	0.579
INQoL	0.001	-0.587	0.001	-0.633
Respiratory tests				
CVF seated	0.012	0.454	0.469	0.140
CVF lying	0.05	0.498	0.666	0.121
MIP	0.246	-0.308	0.254	-0.314
MEP	0.149	-0.392	0.670	0.125

Table 3 shows correlation between MRC Score and myometry score with demographic data, muscle function tests and daily live activities.

### Muscle MRI analysis: T1w imaging and pattern description

**Fig 2:**
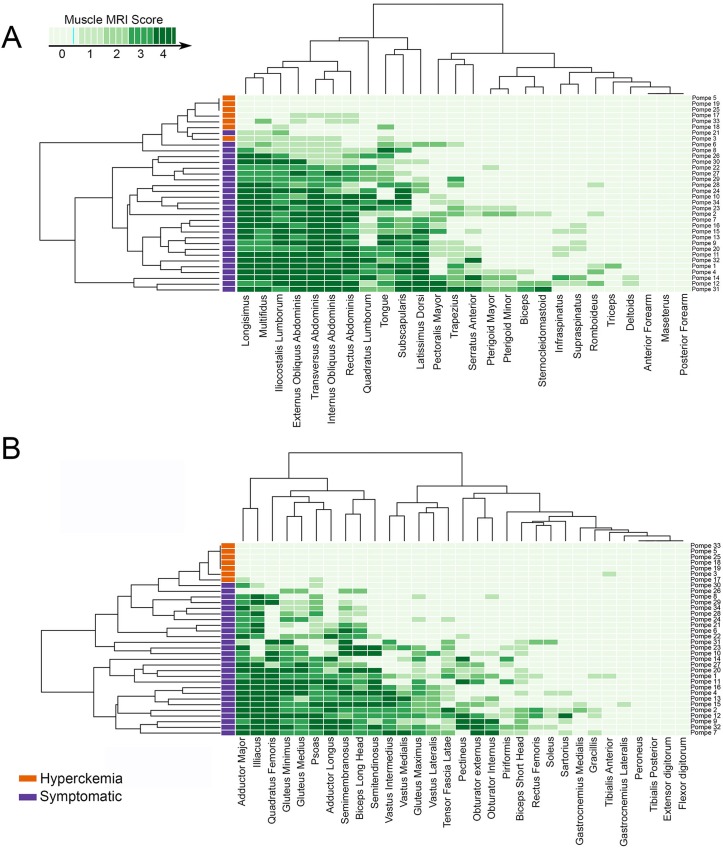
Representation of score of muscle infiltration observed in T1 imaging in each muscle of every patient by heatmaps. (A) Heatmap including muscles of the head, upper limbs and trunk. (B) Heatmap including muscles of the pelvis and lower limbs. In each heatmap, patients (rows) are ordered according to hierarchical clustering with increasing grading in infiltration severity from the top to the bottom. Muscles (columns) are ordered according to dendrogram (upper part of the figures). The score of a muscle in a patient is indicated by the colour of the square in the interaction between the patient and that muscle. The darker the square, the more intense the fatty infiltration of that muscle is.

**Fig 3:**
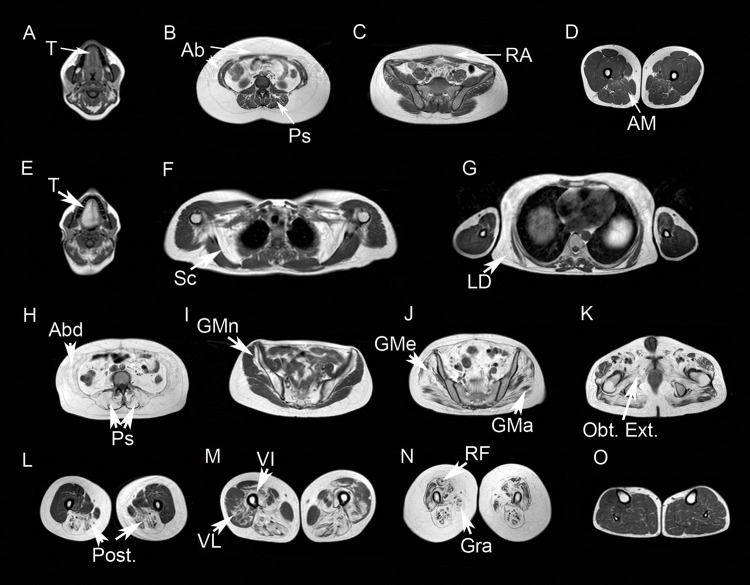
T1w Muscle MRI of patients with Pompe disease. We observed mild fatty infiltration in muscles of hyperCKemia patients: Tongue (Tin A), paraspinal muscles (Ps in B), lateral abdominal muscles (Ab in B) and *rectus abdominis* (RA in C) and in *adductor major* muscles (AM in D). The fatty infiltration in symptomatic patients was more evident (E-L). The muscles more commonly involved were tongue (T in E), *subscapularis* (Sc in F), *latissimus dorsi* (LD in G), the abdominal (Ab in H) and paraspinal muscles (Ps in H), the *Gluteus minor* (GMn in I), *medius* (GMe in J) and *maximus* (GMa in J), the muscles of the pelvic floor such as the *Externus Obturator* (Obt. Ext. in K), the posterior muscles of the thighs (Post in L), and the vasti muscles including *vastus intermedius* (VI in M) and *vastus lateralis* (VL in M). We observed involvement of *rectus femoris* and *gracillis* in advanced patients (RF and Gra in N). Muscles of the lower legs were commonly spared (O).

We performed a hierarchical clustering analysis of values obtained from the quantification of T1w muscle MRI and visualized these data using heatmaps (Fig 2). Our analysis showed predominant involvement of paraspinal muscles, abdominal muscles, tongue, *subscapularis* and *latissimus dorsi* in head and trunk. Head muscles (except tongue), upper limbs and periscapular muscles (except *subscapularis*) were not generally involved. In lower limbs, *glutei* muscles, *psoas*, *illiacus* and posterior muscles of the thighs (*semitendinosus*, *semimembranosus*, *adductor major* and *longus*, and both heads of *biceps*) were predominantly involved. *Glutei minor* and *medius* were more involved than *glutei maximus*. *Vastus intermedius* was the most commonly involved quadriceps muscle, while *rectus femoris* was rarely involved. In contrast, *sartorius* and *gracillis muscles* and lower legs muscles were commonly spared (Fig 2). We show some examples of muscle involvement in Fig 3.

We observed subtle changes in 4 of the 7 pre-symptomatic patients (Fig 2). Mild fatty infiltration (score = 1) was observed in paraspinal muscles (*multifidus*, *longisimus*, *iliocostalis*), abdominal muscles (*rectus abdominis*, *obliquus internus*, *obliquus externus*, *transversus abdominalis*), tongue, and *adductor major* muscles.

### Muscle MRI analysis: 3-point Dixon imaging.

**Fig 4:**
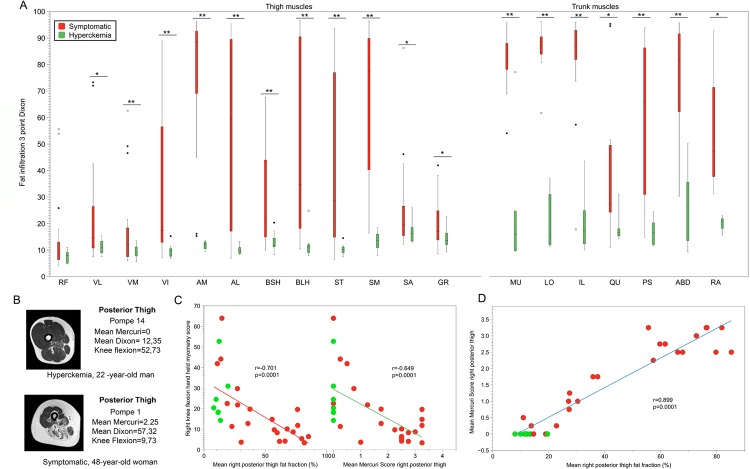
Analysis of fatty infiltration in muscle MRIs. (A) We observed significant differences in the percentage of fatty infiltration quantified using 3-point Dixon technique in most of the muscles between symptomatic (red bars) and hyperckemia patients (green bars). We compared both groups using the Student T test (single asterisk for differences with p<0.05 and double asterisk for differences with p<0.01). (B) Fat-fraction maps of the right thigh in a hyperckemia and a symptomatic AOPD patient. We show the quantification of the fatty involvement of posterior muscles of the thighs using the mean Mercuri Score and the mean 3 point Dixon score (*Semitendinosus*, *Semimembranosus*, *Biceps Long Head and Biceps Short Head)*. Pompe 14 patients (hyperckemia patient) had higher strength in knee flexion measured with the myometer than patient 1 (symptomatic patient). We observed predominant fatty infiltration in the posterior muscles of the thighs producing decreased strength in the knee flexion test. (C) There was a strong correlation between knee flexion strength and the degree of muscle fatty infiltration calculated both using 3-point Dixon (left) and the Mercuri scale (right). Green dots showed values of hyperckemia patients, while red ones represent values of symptomatic patients. (D) We found a strong correlation between 3 point Dixon analysis and Mercuri score. We have represented the correlation between the quantification of fat infiltration using the mean Mercuri score and the mean 3-point Dixon score of the posterior thigh muscles (*Semitendinosus*, *Semimembranosus*, *Biceps Long Head and Biceps Short Head*). RF: *rectus femoris*; VL: *vastus laterallis*; VM: *vastus medialis*; VI: *vastus intermedius*; AM: *adductor major*; AL: *adductor longus*; BSH: *biceps short head*; BLH: *biceps long head*; ST: *semitendinosus*; SM: *semimembranosus*; SA: *sartorius*; GR: *gracillis*; MU: *multifidus*; LO: *longissimus*; IL: *iliocostalis*; QU: *quadratus femoris*; PS: *psoas*; ABD: lateral abdominal muscles; RA: *rectus abdominis*.

We analyzed twelve muscles of the thighs and four muscles of the trunk. Fig 4A summarizes the results, showing significant differences in fatty infiltration of muscles between symptomatic and hyperCKemia patients (Mann-Whitney U test, p<0.05). S1 Fig shows an example of a 3-point Dixon analysis of thighs. The degree of muscle involvement in symptomatic patients varied from severe, as in *Adductor Major* (median fat fraction 78.05%) to mild involvement as in *Rectus femoris* (median fat fraction 13.46%) (S1 Table). Fatty substitution of paraspinal muscles (*multifidus*, *longisimus* and *illiocostalis*) was very high in symptomatic patients, reaching more than 80% in 19/27 symptomatic patients, and was also detectable in hyperCKemia patients (median fat fraction of 22.26%). There was a strong correlation between Mercuri scores detected using T1w imaging and fat fraction analyzed using 3-point Dixon images (Fig 4D).

### Correlation between muscle function tests and muscle MRI

Correlations between myometry measures and fat infiltration in appropriately tested muscles were similar for 3-point Dixon and for T1w imaging (S2 Table). For example, correlation between knee flexion strength and “hamstring average fat fraction” (the average of fatty infiltration in *semimembranosus*, *semitendinosus*, *biceps long* and *short head* muscles) for 3-point Dixon imaging was ρ = -0.70, while for T1w imaging, was r = -0.649 (Fig 4D).

**Fig 5:**
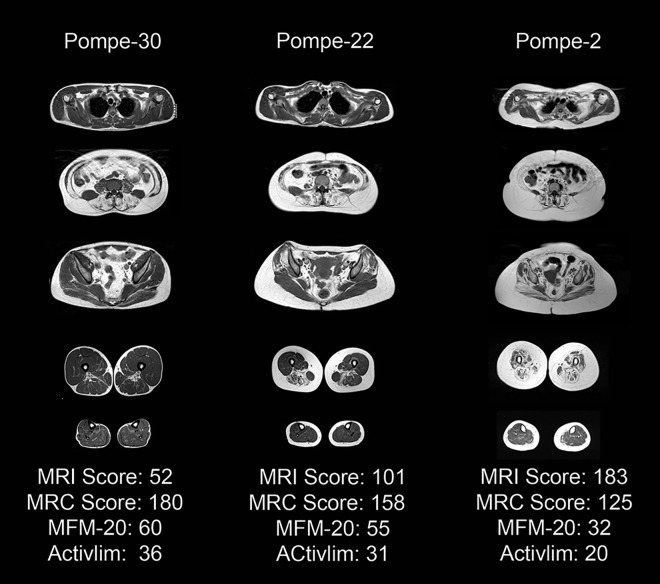
Correlation between Muscle MRI and Functional scales. Patient 30 is a 43 years old man with mild infiltration of pelvic and lower limbs muscles with minor functional impairment. Patient 22 is a 40 years old lady with moderate involvement of trunk, pelvic and thighs muscles. She was able to perform most of her daily live activities with no major problems but her clinical examination showed moderate weakness of pelvic muscles. Patient 2 is a 48 years old lady with a severe involvement of muscle producing severe weakness and notably impairing her daily live activities.

We developed a muscle MRI-T1w score that was the sum of values of the Mercuri scale for each muscle. The mean MRI score was 6 (range: 0 to 15 points) in hyperCKemia patients and 125.9 (range: 39 to 210 points) in symptomatic patients (Table 2). These differences reached statistical significance (Mann-Whitney U, p<0.001). We found strong correlation between muscle MRI-T1w score and most of the functional muscle tests, such as MRC score, 6MWT, MFM-20 or Activlim scale (Table 4). In contrast, we did not find a good correlation with respiratory tests results or quality of life scales (SF-36 and INQoL). Fig 5 shows 3 examples of correlation between Muscle MRI and functional tests.

**Table 4:** Comparison among MRI score and 3-point Dixon correlations with demographic data and muscle function tests.

	T1w-MRI Score		3-point Dixon (Average Thigh)	
	SpearmanTest	Correlation coefficient	Spearman Test	Correlation coefficient
Demographic data				
Gender	0.069	-0.315	0.019	-0.456
Age at MRI	0.055	0.381	0.022	0.391
Time from onset of symptoms	0.257	0.231	0.043	0.349
Delay from onset to ERT	0.421	-0.224	0.498	-0.152
Functional tests					
Myometry Score	0.001	-0.560	0.0001	-0.633
6MWT	0.001	-0.610	0.0001	-0.764
Time to up&go	0.097	0.308	0.006	0.515
Time to climb up 4 stairs	0.0001	-0.782	0.0001	0.858
Time to go down 4 stairs	0.0001	-0.737	0.0001	0.810
MFM-20	0.0001	0.693	0.0001	-0.681
Daily live activities and Quality of life scales					
Activlim	0.0001	0.785	0.0001	-0.754
Respiratory tests					
FVC seated	0.035	-0.386	0.018	-0.469
FVC lying	0.08	-0.450	0.055	-0.544
PIM	0.356	0.247	0.40	0.253
PEM	0.541	-0.171	0.6	-0.16

We also found strong correlation between the average fat fraction of thigh measured using 3-point Dixon imaging and functional scales (Table 4). In most of the cases, correlation coefficients were higher for 3-point Dixon studies than for T1w imaging. For instance, the correlation coefficient between 6MWT (main test used in clinical trials) and 3-point Dixon tests was ρ = -0.764 while it was lower for T1w imaging, r = -0.610. There was no correlation between 3-point Dixon studies and results of respiratory studies.

We analyzed the group of hyperCKemia patients in detail. We found mild changes in muscle MRI in four out of seven cases using T1w imaging that were statistically significant (Mann-Whitney U test, p = 0.008). HyperCKemia patients with changes in MRI were older than 20 years old, whereas patients with no changes were younger than 15 years old. 3-point Dixon analysis showed statistically significant differences in the amount of muscle fatty infiltration of the paraspinal muscles in hyperCKemia patients older than 20 years when they were compared with patients younger than 15 years old (Table 5). In contrast, functional scales were similar in both groups.

**Table 5:** Comparison between presymptomatic patients with hyperckemia depending on the results of the MRI.

	Normal MRI (n = 3))	Abnormal MRI (n = 4))	Mann-Whitney
Age (y)	11	29.2	0.073
MRC Score	180	179.2	0.243
Myometry Score (Nm)	261.24	308.49	0.695
6MWT (min)	616	577	0.572
Time to walk 10 meters (sec)	3.0	3.3	0.571
Time to climb up 4 stairs (sec)	1.4	1.5	0.677
Time to go down 4 stairs (sec)	1.3	1.5	0.455
Time to up&go (sec)	4.8	3.1	0.204
MFM-20	57.6	59.2	0.471
Activlim	36	36	1
T1w-MRI Score	0	10.5	0.008
3-point Dixon Fat fraction Thighs (%)	11.93	11.58	0.865
3-point Dixon Fat fraction Paraspinal muscles (%)	13.54	33.43	0.039

## Discussion

In the present paper we demonstrate that muscle MRI is a useful tool for the study of patients with AOPD for several reasons. Muscle MRI efficiently identifies a group of atrophic muscles that corresponds to the weakest muscles in clinical examination. Muscle MRI has a good correlation with results of the functional muscle scales commonly used in clinical trials. Additionally, muscle MRI has sensitivity to detect early fat infiltration in patients with hyperCKemia before functional scales are impaired. Based on our results, muscle MRI is a acceptable tool to investigate muscle function status in patients with AOPD.

MRI imaging is progressively gaining widespread use to study patients with muscle diseases[14]. There are sequences available to study several aspects of muscle structure. Carlier et al showed the striking utility of using whole-body T1w imaging in a group of twenty AOPD patients, revealing bright signals as indicators of fat replacement in some specific muscles[21]. This technique facilitates pattern recognition when diagnosis is not clear [22]. However, quantification of fatty infiltration using T1w imaging is based usually in visual semiquantitative scales that are observer dependent. On the other hand, analysis of 3-point Dixon images is performed using software that quantifies the exact amount of fat per pixel. Thus, 3-point Dixon is more accurate than T1w imaging to detect slight changes in fatty infiltration. For this reason, 3-point Dixon could be potentially useful in clinical trials of patients with AOPD, in which fatty infiltration slowly progresses over time.

Nevertheless, to consider muscle MRI findings as a reliable outcome measure, it should also correlate with relevant patient function tests[23]. We show strong correlations between muscle MRI results and several functional scales commonly used in clinical trials such as MRC, time to walk 10 meters or the Activlim scale. We detected a significant correlation between muscle strength measured using hand held myometry and muscle fat fraction. Accordingly MRI provides evidence of muscle damage that correlate strongly with muscle strength and function that are independent of participant effort. This fact is especially important in patients with AOPD in whom 6 MWT has been used as the main functional test to analyze response to treatment. 6MWT is not only dependent on muscle strength, but is also influenced by many other factors such as dyspnea and muscle pain, therefore in our opinion, it analyzes general endurance. In fact, we have found a good correlation between muscle MRI and 6MWT. Moreover, the correlation between muscle MRI and muscle strength was strong. Muscle groups that were found atrophic in muscle MRI were the ones weak in MRC study. Heatmaps analyzing MRC (Fig 1) and muscle MRI are practically overlapping (Fig 2). Based on our results, muscle MRI offers a valid and consistent surrogate measure of muscle function. The group of Hovarth studied 7 Pompe patients and 11 controls, using whole-body proton-density fat-fraction imaging[24]. They reported a great sensitivity of MRI to detect subtle changes in skeletal muscles and a good correlation between muscle MRI results and muscle strength analyzed using MRC scale. Our study, which contains a larger number of patients, a more detailed physical examination, a comparison between quantitative and qualitative MRI sequences, and a shorter MRI protocol time (no more than 45 min), confirms their results.

Muscle MRI is also able to identify early changes in muscle signal of several neuromuscular disorders. We observed signs of muscle fatty infiltration in 4 out of 7 patients without any clinical symptom of muscle weakness. Clinical examination, functional and respiratory tests were all normal in these patients. In contrast, muscle MRI already revealed subtle changes, preferentially involving paraspinal and abdominal muscles, suggesting that the process of muscle degeneration had started. A recent retrospective study in a small cohort of AOPD patients analyzing muscle MRI of lower limbs in four asymptomatic patients showed a remarkable increase in fat infiltration of *Adductor magnus* muscle and mild changes in the remaining thigh muscles in one pre-symptomatic patient, suggesting that progressive fatty muscle infiltration starts before clinical manifestations[21]. However, the authors only used T1w imaging to determine mean gray values, which is not a quantitative technique. We have used 3-point Dixon sequences which calculate the exact percentage of fat in every muscle[25]. Moreover, we have analyzed muscle function in detail using several different tests demonstrating the lack of symptoms in patients with isolated hyperCKemia and therefore strengthening our results. In our opinion, detection of a progressive increase in the amount of fatty infiltration in skeletal muscles could potentially be taken into account before deciding to start ERT treatment in a patient with clinically asymptomatic AOPD. Although it is not know how much fatty infiltration in a single muscle is needed to produce weakness, it seems clear that the process of muscle fatty degeneration is not reversible once the treatment is started[26, 27]. Based on our results, it makes sense to follow-up AOPD patients with repeated muscle MRIs, especially those with asymptomatic hyperCKemia. Analysis of paraspinal and abdominal area is crucial to detect changes and to determine whether fatty involvement of muscles progress[28]. We have already described the early involvement of paraspinal muscles in AOPD patients, which can sometimes present as a pure axial myopathy[28]. In this sense, 3-point Dixon imaging technique has emerged as a powerful tool, superior to T1w imaging, to detect subtle changes in percentage of muscle fatty infiltration in skeletal muscles[29]. Other quantitative sequences, such as quantitative T2-mapping, also showed a better profile than T1-weighted imaging to determine minimal changes in AOPD progression[30]. Although our aim was not to analyze disease progression, those results reinforce the need to improve our knowledge and use of new quantitative techniques.

In the last years several groups have started using quantitative MRI sequences to follow-up AOPD patients. However, these new quantitative techniques have some drawbacks: they are not available in many of the medical centers, they need specific software for quantification and the acquisition and analysis of the images consume a lot of time. In order to surpass these limitations, some authors have quantified fat tissue in skeletal muscles of T1w images using imaging software. For example, the group of Dr. Pichiechio followed-up 9 AOPD patients for six-months and the group of Dr. Kley analyzed muscle MRI of 7 AOPD using this technology[31, 32]. Although they were able to identify changes in fat tissue present in the muscles, it has reported than 3-point Dixon is more precise and reliable than T1w analysis for evaluation of fat fractions in longitudinal follow-up studies of patients with neuromuscular disorders[29]. We are currently conducting a prospective study of a large cohort of AOPD patients using 3-point Dixon as a tool in patient follow-up. We hope this new study will further support the usefulness of MRI for routine clinical practice.

In conclusion, muscle MRI is a reliable, valid and surrogate biomarker of muscle function in patients with AOPD. Muscle MRI is not only useful for diagnosis but also may be useful to predict muscle function in these patients. Furthermore, muscle MRI visualizes fatty muscle infiltration in asymptomatic Pompe patients before functional tests are impaired and potentially help to decide when ERT treatment should be started. Based on our observations, we consider muscle MRI could be used as a complementary test in clinical trials and in daily clinics of patients with AOPD.

## Supporting Information

S1 Fig Examples of Fat fraction estimation. (A) Muscles of the thighs can be clearly identified in a single slice. (B) Selection of the region of interest (ROI) in the *vastus laterallis* muscle for the analysis of fat fraction that in this case is of 23.1%. (C) Paraspinal muscles can be identified in a single slice. (D) Selection of the region of interest (ROI) in the *multifidus* muscle for the analysis of fat fraction that in this case is of 21%.
(TIF)

S1 Materials Muscles studied using Fischer modified Mercuri Scale and 3 point Dixon. (DOC)

S1 Table Quantification of fatty muscle infiltration using 3-point Dixon imaging. Quantification of fatty muscle infiltration in muscles of thighs and trunk in symptomatic AOPD patients and patients with hyperckemia only. Student T test was used to compare both groups of patients. P values lower than 0.05 were considered significant.
(DOCX)

S2 Table Comparison of the correlation between quantification of fatty involvement analyzed using T1 imaging and 3-point Dixon with muscle strength in appropriately tested muscles. We correlated the strength of the thighs muscles with the degree of fatty infiltration in muscles analyzed using T1w and 3-point Dixon imaging. We correlated hip flexion with fatty infiltration of psoas muscle, hip adduction with an average of fatty infiltration of *adductor longus* and major, knee extension with the average of fatty infiltration of *rectus femoris*, *vastus medialis*, *vastus intermedius* and *vastus lateralis*; and knee flexion with the average of fatty infiltration of *semitendinosus*, *semimembranosus*, *biceps long head* and *biceps short head*.
(DOCX)
