# Supplementary figures and images for: Muscle MRI Findings in Childhood/Adult Onset Pompe Disease Correlate with Muscle Function
Source: PLoS One. 2016 Oct 6;11(10):e0163493. doi: 10.1371/journal.pone.0163493 (PMC5053479; doi:10.1371/journal.pone.0163493)

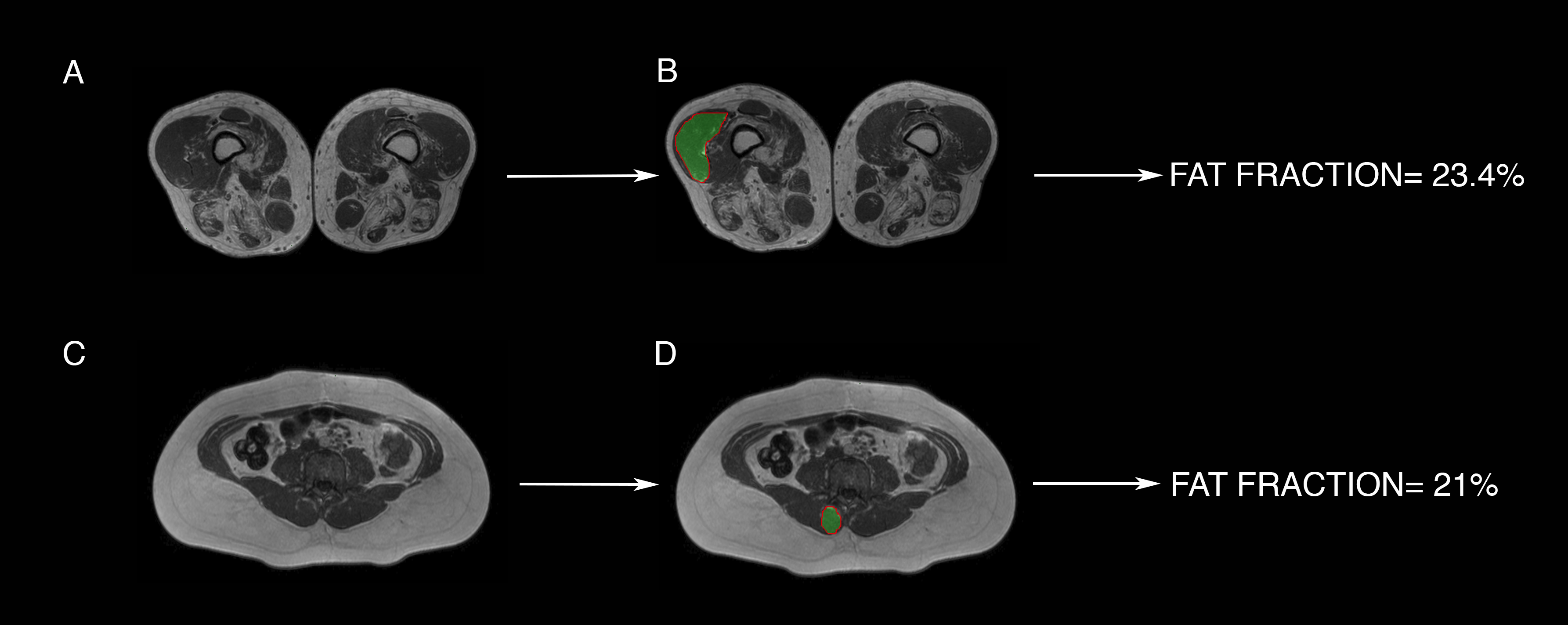

Supplement: S1 Fig — (A) Muscles of the thighs can be clearly identified in a single slice. (B) Selection of the region of interest (ROI) in the vastus laterallis muscle for the analysis of fat fraction that in this case is of 23.1%. (C) Paraspinal muscles can be identified in a single slice. (D) Selection of the region of interest (ROI) in the multifidus muscle for the analysis of fat fraction that in this case is of 21%. (TIF) [file pone.0163493.s001.tif]
