# Supplementary material for: Muscle MRI Findings in Childhood/Adult Onset Pompe Disease Correlate with Muscle Function
Source: PLoS One. 2016 Oct 6;11(10):e0163493. doi: 10.1371/journal.pone.0163493 (PMC5053479; doi:10.1371/journal.pone.0163493)
Supplement: S1 Materials — (DOC) [file pone.0163493.s002.doc]

**Supplemental matherial & methods**

**Muscles analyzed in T1w scans using Mercuri Scale:**

1. Head and neck: *tongue, pterigoid major, pterigoid minor, sternocleidomastoid, masetereus.*
2. Upper limbs: *Deltoids, biceps, triceps, trapezius, latissimus dorsi, supraspinatus, infraspinatus, rhomboideus, subscapularis, pectoralis major, serratus anterior, anterior forearm, posterior forearm.*
3. Trunk and abdominal muscles: *multifidus, longissimus, illiocostalis lumborum, quadratus lumbaris, rectus abdominis, internus obliquus abdominis, externus obliquus abdominis, transversus abdominis, psoas, illiacus.*
4. Pelvic mucles*: gluteus minimus, gluteus medius, gluteus maximus, quadratus femoris, tensor fascia latae, piriformis, pectineus, obturator externus, obturator internus.*
5. Thighs: *vastus intermedius, vastus laterallis, vastus medialis, rectus femoris, Sartorius, gracillis, semitendinosus, semimembranosus, biceps long and short head, adductor major, adductor longus.*
6. Legs: *tibialis anterior, tibialis posterior, peroneus longus, peroneus brevis, gastrocnemius medialis, gastrocnemius laterallis, soleus, flexor digitorum, extensor digitorum.*

**Muscles analysed in 3 point Dixon studies:**

1. Thighs: *vastus intermedius, vastus laterallis, vastus medialis, rectus femoris, Sartorius, gracillis, semitendinosus, semimembranosus, biceps long and short head, adductor major, adductor longus.*
2. Trunk and abdominal muscles: *multifidus, longissimus, illiocostalis lumborum, quadratus lumbaris, rectus abdominis, internus obliquus abdominis, externus obliquus abdominis, transversus abdominis, psoas, illiacus.*
