# Supplementary material for: Muscle MRI Findings in Childhood/Adult Onset Pompe Disease Correlate with Muscle Function
Source: PLoS One. 2016 Oct 6;11(10):e0163493. doi: 10.1371/journal.pone.0163493 (PMC5053479; doi:10.1371/journal.pone.0163493)
Supplement: S1 Table — Quantification of fatty muscle infiltration in muscles of thighs and trunk in symptomatic AOPD patients and patients with hyperckemia only. Student T test was used to compare both groups of patients. P values lower than 0.05 were considered significant. (DOCX) [file pone.0163493.s003.docx]

**Sup. Table 1: Quantification of fatty muscle infiltration using 3-point Dixon imaging**

| **Muscle** | **Symptomatic patients** | **HyperCKemia** | **Student T test** |
| --- | --- | --- | --- |
| *Rectus femoris* | 13.46 (+/- 13.6) | 7.46 (+/- 2.52) | 0.051 |
| *Vastus Lateralis* | 16.67 (+/- 14.91) | 9.46 (+/- 2.80) | 0.034 |
| *Vastus Medialis* | 22.52 (+/- 18.19) | 11.22 (+/- 3.07) | 0.07 |
| *Vastus intermedius* | 33.91 (+/- 27.79) | 9.76 (+/- 2.91) | 0.0001 |
| *Adductor longus* | 52.77 (+/- 35.94) | 10.10 (+/- 1.82) | 0.0001 |
| *Adductor major* | 78.05 (+/- 23.37) | 11.80 (+/- 1.61) | 0.0001 |
| *Semimembranosus* | 65.04 (+/- 28.71) | 13.40 (+/-3.72) | 0.0001 |
| *Semitendinosus* | 43.09 (+/-32.73) | 10.38 (+/-2.23) | 0.0001 |
| *Biceps Long Head* | 50.24 (+/- 34.72) | 12.34 (+/- 5.76) | 0.0001 |
| *Biceps Short Head* | 30.12 (+/- 18.19) | 13.10 (+/- 4.14) | 0.0001 |
| *Sartorius* | 24.59 (+/- 15.81) | 17.07 (+/- 4.69) | 0.048 |
| *Gracillis* | 20.86 (+/- 9.68) | 14.67 (+/- 4.63) | 0.029 |
| *Multifidus* | 82.39 (+/- 9.43) | 25.55 (+/- 25.03) | 0.003 |
| *Longisimus* | 86.93 (+/- 7.23) | 20.45 (+/- 10.97) | 0.0001 |
| *Iliocostalis* | 83.16 (+/- 17.95) | 20.94 (+/- 12.27) | 0.0001 |
| *Quadratus Lumbaris* | 41.98 (+/- 29.48) | 19.09 (+/- 6.84) | 0.031 |
| *Psoas* | 58.13 (+/- 30.22) | 16.89 (+/- 5) | 0.0001 |
| *Abdominal muscles* | 73.71 (+/- 22.09) | 27 (+/- 14.85) | 0.0001 |

Quantification of fatty muscle infiltration using 3-point Dixon imaging

Quantification of fatty muscle infiltration in muscles of thighs and trunk in symptomatic AOPD patients and patients with hyperckemia only. Student T test was used to compare both groups of patients. P values lower than 0.05 were considered significant
