# Supplementary material for: Muscle MRI Findings in Childhood/Adult Onset Pompe Disease Correlate with Muscle Function
Source: PLoS One. 2016 Oct 6;11(10):e0163493. doi: 10.1371/journal.pone.0163493 (PMC5053479; doi:10.1371/journal.pone.0163493)
Supplement: S2 Table — We correlated the strength of the thighs muscles with the degree of fatty infiltration in muscles analyzed using T1w and 3-point Dixon imaging. We correlated hip flexion with fatty infiltration of psoas muscle, hip adduction with an average of fatty infiltration of adductor longus and major, knee extension with the average of fatty infiltration of rectus femoris, vastus medialis, vastus intermedius and vastus lateralis; and knee flexion with the average of fatty infiltration of semitendinosus, semimembranosus, biceps long head and biceps short head. (DOCX) [file pone.0163493.s004.docx]

**Sup. Table 2: Comparison of the correlation between quantification of fatty involvement analyzed using T1 imaging and 3-point Dixon with muscle strength in appropriately tested muscles.**

| **Muscles** | **T1w imaging** | | **3-point Dixon** | |
| --- | --- | --- | --- | --- |
|  | Spearman Test | Correlation coefficient | Spearman test | Correlation coefficient |
| Hip flexion | 0.077 | -0.360 | 0.083 | -0.311 |
| Hip adduction | 0.012 | -0.437 | 0.0001 | -0.654 |
| Knee extension | 0.002 | -0.517 | 0.0001 | -0.631 |
| Knee flexion | 0.0001 | -0.649 | 0.0001 | -0.70 |

We correlated the strength of the thighs muscles with the degree of fatty infiltration in muscles analyzed using T1w and 3-point Dixon imaging. We correlated hip flexion with fatty infiltration of *psoas* muscle, hip adduction with an average of fatty infiltration of *adductor longus* and *major*, knee extension with the average of fatty infiltration of *rectus femoris*, *vastus medialis*, *vastus intermedius* and *vastus lateralis*; and knee flexion with the average of fatty infiltration of *semitendinosus*, *semimembranosus*, *biceps long head* and *biceps short head*.
